# Supplementary material for: Visual assessment of dynamic knee joint alignment in patients with patellofemoral pain: an agreement study
Source: PeerJ. 2021 Oct 19;9:e12203. doi: 10.7717/peerj.12203 (PMC8532969; doi:10.7717/peerj.12203)
Supplement: Supplemental Information 1 [file peerj-09-12203-s001.pdf]

Supplementary file for manuscript: Visual assessment of dynamic knee joint alignment in patients with patellofemoral pain: an agreement study

Table 1. Intrarater agreement Forward Lunge - raw scores

		rater 1									
		**-4**	**-3**	**-2**	**-1**	**0**	**1**	**2**	**3**	**4**	**Total**
**rater 1**	**-4**	1	2	0	0	0	0	0	0	0	3
	**-3**	0	6	2	0	0	0	0	0	0	8
	**-2**	0	3	4	4	1	0	0	0	0	12
	**-1**	0	1	5	10	2	0	0	0	0	18
	**0**	0	0	1	3	15	0	0	0	0	19
	**1**	0	0	0	0	0	0	0	0	0	0
	**2**	0	0	0	0	0	0	0	0	0	0
	**3**	0	0	0	0	0	0	0	0	0	0
	**4**	0	0	0	0	0	0	0	0	0	0
	**Total**	1	12	12	17	18	0	0	0	0	60

Table 2. Intrarater agreement Forward Lunge – classification

		rater 1			
		**Varus**	**Neutral**	**Valgus**	**Total**
**rater 1**	**Varus**	0	0	0	0
	**Neutral**	0	30	7	37
	**Valgus**	0	5	18	23
	**Total**	0	35	25	60

Table 3. Interrater agreement Forward Lunge - raw scores

		rater 2									
		**-4**	**-3**	**-2**	**-1**	**0**	**1**	**2**	**3**	**4**	**Total**
**rater 1**	**-4**	0	3	0	0	0	0	0	0	0	3
	**-3**	0	4	3	1	0	0	0	0	0	8
	**-2**	0	2	4	6	0	0	0	0	0	12
	**-1**	0	0	5	10	3	0	0	0	0	18
	**0**	0	1	2	5	10	1	0	0	0	19
	**1**	0	0	0	0	0	0	0	0	0	0
	**2**	0	0	0	0	0	0	0	0	0	0
	**3**	0	0	0	0	0	0	0	0	0	0
	**4**	0	0	0	0	0	0	0	0	0	0
	**Total**	0	10	14	22	13	1	0	0	0	60

Table 4. Interrater agreement Forward Lunge – classification

		rater 2			
		**Varus**	**Neutral**	**Valgus**	**Total**
**rater 1**	**Varus**	0	0	0	0
	**Neutral**	0	29	8	37
	**Valgus**	0	7	16	23
	**Total**	0	36	24	60

Table 5. Intrarater agreement single leg squat - raw scores

		rater 1									
		**-4**	**-3**	**-2**	**-1**	**0**	**1**	**2**	**3**	**4**	**Total**
rater 1	**-4**	5	1	0	0	0	0	0	0	0	6
	**-3**	3	11	4	1	0	0	0	0	0	19
	**-2**	0	4	8	4	0	0	0	0	0	16
	**-1**	0	0	2	6	2	0	0	0	0	10
	**0**	0	0	1	4	3	1	0	0	0	9
	**1**	0	0	0	0	0	0	0	0	0	0
	**2**	0	0	0	0	0	0	0	0	0	0
	**3**	0	0	0	0	0	0	0	0	0	0
	**4**	0	0	0	0	0	0	0	0	0	0
	**Total**	8	16	15	15	5	1	0	0	0	60

Table 6. Intrarater agreement single leg squat - classification

		rater 1			
		**Varus**	**Neutral**	**Valgus**	**Total**
rater 1	**Varus**	0	0	0	0
	**Neutral**	0	16	3	19
	**Valgus**	0	5	36	41
	**Total**	0	21	39	60

Table 7. Interrater agreement single leg squat - raw scores

		rater 2									
		**-4**	**-3**	**-2**	**-1**	**0**	**1**	**2**	**3**	**4**	**Total**
rater 1	**-4**	1	3	1	0	1	0	0	0	0	6
	**-3**	2	9	4	4	0	0	0	0	0	19
	**-2**	0	5	4	7	0	0	0	0	0	16
	**-1**	0	1	4	4	1	0	0	0	0	10
	**0**	0	0	4	2	2	1	0	0	0	9
	**1**	0	0	0	0	0	0	0	0	0	0
	**2**	0	0	0	0	0	0	0	0	0	0
	**3**	0	0	0	0	0	0	0	0	0	0
	**4**	0	0	0	0	0	0	0	0	0	0
	**Total**	3	18	17	17	4	1	0	0	0	60

Table 8. Interrater agreement single leg squat - classification

		rater 2			
		**Varus**	**Neutral**	**Valgus**	**Total**
rater 1	**Varus**	0	0	0	0
	**Neutral**	0	10	9	19
	**Valgus**	0	12	29	41
	**Total**	0	22	38	60
